# Assessing knowledge, attitude, practice, and preparedness of hospital pharmacists in Lebanon towards COVID-19 pandemic: a cross-sectional study

**DOI:** 10.1186/s40545-020-00266-8

**Published:** 2020-09-18

**Authors:** Rony M. Zeenny, Elsy Ramia, Youssef Akiki, Souheil Hallit, Pascale Salameh

**Affiliations:** 1 Pharmacy Department, American University of Beirut Medical Center, Beirut, Lebanon; 2 School of Pharmacy, Lebanese American University, Byblos, Lebanon; 3 Faculty of Pharmacy, Lebanese University, Hadat, Lebanon; 4 Faculty of Medicine and Medical Sciences, Holy Spirit University of Kaslik (USEK), Jounieh, Lebanon; 5 INSPECT-LB, Institut National de Santé Publique, Épidémiologie Clinique et Toxicologie-Liban, Beirut, Lebanon; 6 Faculty of Medicine, Lebanese University, Hadat, Lebanon

**Keywords:** COVID-19, Knowledge, Attitudes, Practice, Emergency preparedness, Middle East, Lebanon, Assessment of healthcare needs, Hospital pharmacy services, Rony M Zeenny and Elsy Ramia are co-first authors.

## Abstract

**Background:**

During the COVID-19 pandemic, the Lebanese government has taken the proactive anticipatory measure to minimize the infection rates. Despite the pivotal role of the pharmacists working in hospital settings, hospital pharmacists have not been engaged in the emergency preparedness for hospitals. The primary objective of this survey is to assess the knowledge, attitude, and practice of hospital pharmacists in Lebanon towards COVID-19 pandemic and explore the level of health emergency preparedness of Lebanese hospitals in response to this outbreak.

**Methods:**

A standardized English-based, anonymous and online questionnaire was diffused via social media platforms to all Lebanese hospital pharmacists. The questionnaire consisted of 78 questions related to sociodemographic characteristics, knowledge-based, attitude-based, practice questions, and pandemic preparedness. Descriptive statistical analysis was used to summarize data.

**Results:**

A total of 81 questionnaires were completed; the participants were able to know > 90% of the knowledge-based questions regarding COVID-19. Most of the respondents were concerned about getting infected and their families due to their professional exposure. Similarly, around 67% were following the safety recommendations. Most of the participants agreed that they are facing shortages, rising prices, and delays in supply of masks and sanitizers. In terms of COVID-19 readiness, about 50% of hospitals have taken practical steps.

**Conclusions:**

Our findings revealed an appropriate level of knowledge and good practice towards COVID-19, among the respondents from Lebanese hospitals. National organizations may benefit in utilizing the expertise of the hospital pharmacists to be able to minimize/avoid future waves of COVID-19 if it emerges.

## Background

The COVID-19 pandemic has posed a significant health and economic challenge for the global and development of the community [1]. In less than a month, the global number of confirmed COVID-19 cases doubled from about 75,000 cases on February 20 to more than 153,000 on March 15 to reach 6,400,000 on June 2 [1, 2]. That infection rate, as scary as it sounds, hides just how much the out-of-control virus has spread, especially in the hardest-hit communities. Efforts to completely contain the new coronavirus, responsible for infecting people in 213 countries and territories [2], have failed. Although the profile of the disease suggests that 80% of cases are mild, some 15% of people react severely while 5% become severely ill (septic shock, respiratory, and organ failure) [3]. Severe cases affect patients with underlying conditions such as cardiovascular disease, hypertension, and diabetes or elderly; older age is a particular concern in many countries with aging populations [3]. Transmission can occur from person to person by droplets from an infected individual or contaminated surfaces or objects [4].

No known effective treatment for COVID-19 virus exists [5]; therefore, management is largely intensive supportive care [5]. Many experimental therapies including existing anti-infective agents, including antibiotics, antivirals, and antiparasitics, in addition to antibody therapy are being studied and may be useful in the future [5]. New molecules also continue to be evaluated and tested as potential future antiviral therapies against the COVID-19 virus and others [5]. The most impactful intervention would be the implementation of an effective vaccine during an outbreak event. Many COVID-19 vaccine candidates are currently being studied in early phase trials. As there is currently no vaccine or specific medication to treat COVID-19 [6], and because testing is so limited, the only way to flatten the curve [7] is through collective action. The US Centers for Disease Control and Prevention (CDC) has recommended that all Americans wash their hands frequently, self-isolate when they are sick or suspect they might be, and start “social distancing” (essentially, avoiding other people whenever possible) right away [8].

Governments across the world have responded to the pandemic with a set of actions including closures of schools and public attractions, implementation of travel bans, quarantines, and nationwide lockdowns [9]. Sequentially, the COVID-19 pandemic has created an unprecedented disruption of the economy [10].

In general, emergencies put health systems and their ability to deliver health care services under strain. Currently, health care services in the various regions around the world are being confronted with increased demand generated by the COVID-19 outbreak [11]. When health systems are overwhelmed, morbidity is exacerbated, disability intensifies, and both mortality from the outbreak and mortality from vaccine-preventable and treatable conditions increase [12]. Responding exclusively to COVID-19 cases, without considering how the delivery of essential health care services will be maintained across the continuum of care from prevention to palliation, comes with several risks [12]. Organizations fighting infectious diseases, supporting health workers and delivering social services, have become the center of attention. But their work is complicated by a set of challenges related to access, safety, supply chain logistics, and financial stress [13].

The COVID-19 pandemic is primarily a health shock [14]. Knowing that the primary objective is health emergency, countries have focused their attention on a set of critical interventions [15].

Several European countries have shared common concerns about the readiness and capacity of their health systems to respond to the novel coronavirus (SARS-CoV-2) COVID-19 pandemic [3].

The American Society of Health-System Pharmacists (ASHP) has highlighted the role of the pharmacist in emergency preparedness, including biological disasters [16], and has made available COVID-19 Pandemic Assessment tools intended to assist pandemic planning efforts in the department of pharmacy and to foster pharmacist involvement in preparedness at the institutional and community level [17]. Other pharmacy organizations such as the International Pharmaceutical Federation (FIP) have dedicated a set of resources to help pharmacists during this pandemic [18]. In fact, pharmacists have an important multifaceted role in disaster preparedness and disaster response. The American Pharmacists Association (APhA) mentioned in its Policy Manual disaster preparedness section published in 2015 that the APhA “encourages pharmacist involvement in surveillance, mitigation, preparedness, planning, response, and recovery related to bioterrorism and emerging infectious diseases” [19]. Pharmacists, being the medication experts [20], should lead in managing medication supply chains and distribution systems such as procurement, storage, compounding, and dispensing in emergencies [16, 19, 21]. Furthermore, pharmacist expertise will help in guiding treatment decisions and drug information questions for the most vulnerable individuals including children, elderly, pregnant women, and immunocompromised patients, as well as developing local treatment plans. Besides, pharmacists possess the ability to provide direct patient care by medication therapy management, adherence counseling, and monitoring (particularly for the long durations of PEP) [1, 22]. This is particularly true for hospital pharmacists. In Lebanon, the government and the Ministry of Public Health (MOPH) have taken the proactive anticipatory decision of closure and were able to raise awareness to keep the numbers of patients infected with COVID-19 to the minimal [23]. As of June 2, 2020, the total number of confirmed cases is 1242 with 27 deaths overall. The majority of the cases are males (59%), while only 16.8% are above 60 years of age. Interestingly, 57.5% of the cases had mild/moderate presentation, 37% had no symptoms at all, in contrast to 5.5% who had severe conditions [23]. Luckily, the cases reported to date did not overwhelm the Lebanese health system, including private (12,749 beds, 1020 respirators) and public hospitals (2446 beds, 165 respirators) [24].

Despite the preventive measures taken on national levels, there has been no guidance or support or involvement in emergency preparedness for hospital pharmacists on how to deal with such a pandemic. The Order of Pharmacists of Lebanon has only issued minimal guidance for pharmacists working in a community setting [25]. Therefore, the main objective of this survey is to assess the knowledge, attitude, and practice of hospital pharmacists in Lebanon towards COVID-19 pandemic and explore the level of health emergency preparedness of Lebanese hospitals in response to this pandemic.

## Methods

### Study design and procedure

This was a descriptive cross-sectional observational study based on an online anonymous survey. It was conducted from March 29, 2020, through May 10, 2020. The voluntary survey was carried out on licensed pharmacists working in hospital pharmacies located in all Governorates of Lebanon (Beirut, Mount Lebanon, North, South, and Bekaa). To minimize interview risks as well as the lockdown restrictions enforced by the Lebanese Government, a snowball sampling method was used as an approach to the survey using online Google forms [26]. The survey was distributed via social applications including WhatsApp, LinkedIn, and Facebook. All invited participants were above 18 years of age. Licensed pharmacists practicing in other settings or not currently working in Lebanon were excluded from this survey.

### Sample size

The sample size was determined by using Epi Info 7 StatCalc functions for a population survey. Considering that at the time of the survey, 320 licensed pharmacists were working in hospital settings, with an expected confidence level of 95%, a margin of error of 5%, and assuming that 90% of the hospital pharmacists were familiar with the recommendations of the ASHP [15] that were circulated on the hospital pharmacists group and were available online, the minimal sample size was found to be 75.

### Survey development

A group of 5 pharmacists with extended experience in hospital pharmacy and academia developed the questionnaire based on a thorough review of the literature [3, 4, 8, 18, 23, 25] and the latest recommendations of professional organizations. The preparedness questions were adapted from the ASHP COVID-19 pandemic assessment tool for health-system pharmacy departments [17]. The questionnaire was piloted and reviewed by another set of 5 hospital pharmacists. All questions were simplified for better understanding (Additional file 1). The final questionnaire required an average of 14 min to complete.

The questionnaire was developed in English language and consisted of 78 questions using a combination of Likert scales and multiple choice questions. It is composed of the following sections: sociodemographic characteristics (1–14), knowledge (15–27), attitude (28–34), practice (35–57), and pandemic planning efforts and preparedness (58–78). It also included a free-text section at the end where the participant could add any suggestion or comment. The answers provided by the pharmacists were collected on an online sheet.

### Data management and statistical analysis

Statistical analyses were performed using the Statistical Package for the Social Sciences for Windows, Version 23.0 (SPSS Inc. - IBM Corp., Armonk, NY, USA). Descriptive statistics were calculated. Means and standard deviations were reported for continuous variables. Categorical variables were assessed and described as frequency and percentage.

## Results

### General characteristics of hospital pharmacists

A total of 81 hospital pharmacists were included in the survey (mean age 39.36 years ± SD 10.48, 85.2% females). The highest percentage of respondents was from Beirut. The great majority had a PharmD or Master’s degree (80.2%), while only 19.8% had a BS in Pharmacy (Table 1).

**Table 1:** Characteristics of Lebanese hospital pharmacist

Characteristics of the pharmacist	Total = 81
Age (mean ± SD)	39.36 ± 10.48
Gender	
Males	12 (14.8%)
Females	69 (85.2%)
Mouhafazat	
Beirut	35 (43.2%)
Mount Lebanon	25 (30.9%)
North	9 (11.1%)
South	5 (6.2%)
Bekaa	7 (8.6%)
Educational background	
Bachelor in Science (BS) in Pharmacy (only)	16 (19.8%)
BS in Pharmacy + master degree	14 (17.3%)
Pharmacy doctor (Pharm.D.)	51 (62.9%)
Years of experience in hospital pharmacy (mean ± SD)	11.56 ± 8.24
Job title/position	
Associate-assistant director/chief	5 (6.2%)
Clinical pharmacists/staff pharmacist	25 (30.9%)
Chief pharmacist/director	51 (63.0%)
Employment status	
Part-timer	1 (1.2%)
Full timer	80 (98.8%)
Hospital location	
Rural/peripheral area	15 (18.5%)
Urban area	66 (81.5%)
Number of beds per hospital	
25 beds or less	5 (6.2%)
26–99 beds	25 (30.9%)
100–199 beds	22 (27.2%)
200–299 beds	8 (9.9%)
300–499 beds	18 (22.2%)
500 or more beds	3 (3.7%)
Type of hospital	
Governmental; non-teaching hospital	6 (7.4%)
Governmental; teaching hospital	5 (6.2%)
Private; non-teaching hospital	32 (39.5%)
Private; teaching hospital	38 (46.9%)
Number of staff/clinical pharmacists on duty at a single point in time (including respondent)	
1	29 (35.8%)
2	13 (16.0%)
3	8 (9.9%)
4 and more	31 (38.3%)
Number of pharmacy technicians on duty at a single point in time	
0	5 (6.2%)
1	8 (9.9%)
2	8 (9.9%)
3	16 (19.8%)
4 or more	44 (54.3%)
Facility with a physician residency training program	
No	38 (46.9%)
Yes	43 (53.1%)
The facility as a clinical training site to students from an accredited program	
No	18 (22.2%)
Yes	63 (77.8%)

### Knowledge related to COVID-19

Knowledge of hospital pharmacists in Lebanon was appropriate for most simple questions (more than 90%), particularly regarding preventive measures, concentration of alcohol to kill viruses, and medication use (Table 2).

**Table 2:** Knowledge of Lebanese hospital pharmacists related to COVID-19

Questions	*N* = 81(%)
Which of the following is true about COVID-19? *	
▪ **Person to person transmission can occur by droplets** ▪ **Transmission can be airborne** ▪ Most common symptoms include fever, diarrhea, and dyspnea ▪ I do not know	**100%** **33 (40.7%)** 62 (76.5%) 0
What are the steps to take to protect yourself? *	
▪ Wash your hands with soap and water for at least 10 s ▪ **Wash your hands with soap and water for at least 20 s** ▪ **Avoid close contact; put a distance with other people (1.5–2 m)** ▪ **Wear a facemask and stay home if you have any respiratory symptom**	2 (2.5%) **79 (97.5%)** **80 (98.8%)** **74 (91.4%)**
**Which of the below products are you recommending to patients to disinfect?**	
▪ **Alcohol 60%** ▪ **Alcohol 70%** ▪ Alcohol 95%	**13 (16.0%)** **80 (98.8%)** 13 (16.0%)
Indicate which of these options can be used to treat COVID 19 to date? *	
▪ **Acetaminophen** ▪ -steroidal anti-inflammatory drugs (NSAIDs) ▪ Corticosteroids ▪ **Symptomatic respiratory relief (inhalers)** ▪ **Lopinavir/ritonavir (initially for HIV)** ▪ **Chloroquine/remdesivir in combination** ▪ **Tocilizumab (initially for rheumatoid arthritis)**	**69 (85.2%)** 1 (1.2%) 7 (8.6%) **47 (58.0%)** **61 (75.3%)** **29 (35.8%)** **53 (65.4%)**
**Intravenous high dose vitamin C is recommended for COVID 19 treatment?**	
▪ **True** ▪ False ▪ I do not know	**29 (35.8%)** 32 (39.5%) 20 (24.7%)

Correct answers in bold

*More than one answer can be correct

### Information about COVID-19

The majority of pharmacists reported getting information about COVID-19, mainly for 1–2 h per day (48%) and some for less than 1 h (25%). As for sources of information, the most trusted were World Health Organization—WHO (83%), MOPH (74%), and CDC (67%). However, 53% still relied on television, 44% on unspecified websites, 33% on Facebook, and 38.3% on family and friends (Table 3).

**Table 3:** Information about COVID-19

Questions	*N* = 81(%)
**Do you have time to get information regarding the COVID-19 outbreak?**	
▪ 3–4 h/day ▪ 1–2 h/day ▪ < 1 h/day	22 (27.2%) 39 (48.1%) 20 (24.7%)
**Where do you get your information on COVID-19 from?**	
▪ CDC website ▪ Ministry of Public Health website (MOPH) ▪ World Health Organization (WHO) ▪ Infectious Disease Society of America (IDSA) ▪ Media website/Internet ▪ Facebook ▪ Friends/family members ▪ Television ▪ Pharmacists groups ▪ Other, specify: ECDC, LSID, Webinar	54 (66.7%) 60 (74.1%) 67 (82.7%) 29 (35.8%) 53 (44.2%) 27 (33.3%) 31 (38.3%) 43 (53.1%) 4 (4.9%) 3 (3.7%)

### Additional matters related to MOPH hotline and other comments

Very few pharmacists tried to reach the MOPH COVID-19 call center (12.3%), and 69.1% reported having attended an awareness session on COVID-19. In the last open-ended question, some pharmacists expressed many needs, including information and clear recommendations.

### Attitude of Lebanese hospital pharmacists towards COVID-19

The majority of pharmacists were concerned about getting infected and their families due to their professional exposure. More than half of them declared being exhausted because of the pandemic but claimed that their stress did not affect their professional duties nor their family and staff relationships, and a few employees (29%) expressed their desire to quit their jobs. Less than 50% of pharmacies have implemented icebreaking or energizing actions to mitigate staff stress (Table 4).

**Table 4:** Attitude of Lebanese hospital pharmacists towards COVID-19

Questions	Never	Rarely	Often	Always
Are you afraid of getting infected with COVID-19 due to occupational exposure?	2 (2.5%)	27 (33.3%)	35 (43.2%)	17 (21.0%)
Are you afraid your family gets infected due to your occupational exposure?	1 (1.2%)	10 (12.3%)	26 (32.1%)	44 (54.3%)
Do you feel depressed/exhausted due to the current pandemic?	6 (7.4%)	23 (28.4%)	45 (55.6%)	7 (8.6%)
Are stress feelings affecting your duties (counseling, education, assessment)?	20 (24.7%)	38 (46.9%)	20 (24.7%)	3 (3.7%)
Are stress feelings affecting your relationship with your staff and family members?	20 (24.7%)	38 (46.9%)	20 (24.7%)	3 (3.7%)
Does any of your staff declare wanting to leave work due to COVID-19 fear?	42 (51.9%)	16 (19.8%)	16 (19.8%)	7 (8.6%)
Do you implement specific icebreaking or energizing actions in your pharmacy to mitigate your staff stress?	16 (19.8%)	27 (33.3%)	27 (33.3%)	11 (13.6%)

### Practice of hospital pharmacists regarding safety

Two thirds of hospital pharmacists reported following appropriate safety recommendations. As for shifts management, around half of pharmacists are still operating as usual, with no reduction in timing and no change or rotation when shifts overlap. Nevertheless, 8.6% of staff were tested positive for COVID-19 till now. For those wearing masks, they would change it after a median of 5 h; for gloves, it would be after 2.5 h. Although more than 90% declared being able to frequently wash or rub their hands, and avoid touching their face, a lower percentage could keep a safe distance from their work colleagues (80%). About 22% do not stay home even if they are not feeling well, 26% do never wear gloves, and 30% rarely wear masks (Table 5).

**Table 5:** Practice of hospital pharmacists regarding safety

Questions		No	Yes	NA
Was any of your staff members tested positive for COVID-19?		73 (90.1%)	7 (8.6%)	1 (1.2%)
Were training hours suspended during the pandemic?		12 (14.8%)	50 (61.7%)	19 (23.5%)
Are you required to wear a mask while performing your job?		27 (33.3%)	49 (60.5%)	5 (6.2%)
Are you required to wear gloves while performing your job?		45 (55.6%)	32 (39.5%)	4 (4.9%)
Are you wearing goggles/glasses to protect your eyes while performing your job at the pharmacy?		75 (92.6%)	5 (6.2%)	1 (1.2%)
Are you still working as a full team as before COVID-19?		59 (72.8%)	22 (27.2%)	–
If you are alternating schedule, were you asked to use vacation days?		21 (25.9%)	51 (63.0%)	9 (11.1%)
Did the working hours decrease for staff to decrease exposure?		37 (45.7%)	41 (50.6%)	3 (3.7%)
Did the working hours decrease for the chief pharmacist to decrease exposure?		41 (50.6%)	36 (44.4%)	4 (4.9%)
Were staff rotations changed in a way to decrease exposure?		15 (18.5%)	58 (71.6%)	8 (9.9%)
For how long do you put your face mask before changing it (hours) (median) [IQR]?		5 (IQR = 3–7)		
For how long do you put your gloves before changing them (hours) (median) [IQR]?		2.5 (IQR = 1–7)		
Additional questions	Never	Rarely	Often	Always
Are you able to wash your hands during your shift?	0	0	11 (13.6%)	70 (86.4%)
Are you able to rub your hands with hydro-alcoholic gel during your work shift?	1 (1.2%)	0	11 (13.6%)	69 (85.2%)
Are you able to maintain social distancing of at least 1.5 m from work colleagues?	4 (4.9%)	11 (13.6%)	37 (45.7%)	29 (35.8%)
Are you able to avoid touching eyes, nose, and mouth?	0	12 (14.8%)	45 (55.6%)	24 (29.6%)
Are you able to stay home if not feeling well?	6 (7.4%)	12 (14.8%)	25 (30.9%)	38 (46.9%)
Do you put gloves during your work shift?	21 (25.9%)	28 (34.6%)	15 (18.5%)	17 (21.0%)
Do you wear a mask during your work shift?	10 (12.3%)	15 (18.5%)	18 (22.2%)	38 (46.9%)

### Provision of protective devices and supplements

The majority agreed that they had a shortage of masks, gloves, and hand sanitizers. Almost all pharmacists were facing supply delays, rising prices, and pressure from suppliers to pay in cash, and after short periods (Table 6).

**Table 6:** Protective devices supply

Questions about supply	No	Yes	NA
Are you facing any shortage of masks, gloves, and hand gels at your hospital?	23 (28.4%)	56 (69.1%)	2 (2.5%)
Are you facing delays in the supply of masks, gloves, or hand gels from suppliers?	9 (11.1%)	66 (81.5%)	6 (7.4%)
Are you facing an increase in the price of the masks, gloves, and hand gels from the supplier in a regular manner?	5 (6.2%)	68 (84.0%)	8 (9.9%)
Are you facing pressure from suppliers to pay cash or in a short period?	7 (8.6%)	63 (77.8%)	11 (13.6%)

### Practice related to the hospital preparedness for COVID-19

At the time of the survey, almost half of the hospitals had taken practical steps in preparation for COVID-19 pandemic spreading in Lebanon; one quarter to one third were still working on their plans, but some were not taking any measures as institutions (Table 7).

**Table 7:** Practice related to hospital preparedness for COVID-19

Item	Percentage ***N*** = 81(%)
Is your hospital part of the national network to receive COVID-19 patients?	
In progress No Yes	18 (22.2%) 35 (43.2%) 28 (34.6%)
Does your hospital have an emergency preparedness/management committee for COVID-19?	
In progress No Yes	21 (25.9%) 7 (8.6%) 53 (65.4%)
Is the pharmacy represented on the emergency preparedness/management committee of the hospital, and has it participated in the development of the hospital plan?	
In progress No Yes	11 (13.6%) 19 (23.5%) 51 (63.0%)
Is the pharmacist familiar with best practices from other available plans (national, MOPH, and international, WHO and CDC) related to the pharmacy?	
In progress No Yes	23 (28.4%) 5 (6.2%) 53 (65.4%)
Has the pharmacy participated in the development of a hospital infection control plan for managing hospital patients with COVID-19?	
In progress No Yes	16 (19.8%) 22 (27.2%) 43 (53.1%)
In the pharmacy plan, has the pharmacy included a section that describes backup for key pharmacy roles?	
In progress No Yes	23 (28.4%) 25 (30.9%) 33 (40.7%)
Has the pharmacy designated a pharmacy staff member to coordinate education and training on COVID-19?	
In progress No Yes	21 (25.9%) 45 (55.6%) 15 (18.5%)
Has the pharmacy prepared a plan to educate pharmacy staff on infection control measures, social distancing practices, PPE, prophylaxis, and treatment?	
In progress No Yes	26 (32.1%) 16 (19.8%) 39 (48.1%)
Does the pharmacy routinely monitor to ensure that pharmacy staff adheres to infection control and social distancing measures?	
In progress No Yes	23 (28.4%) 10 (12.3%) 48 (59.3%)
Has the pharmacy identified and trained multiple staff members to perform the critical pharmacy activities including administrative, clinical, distribution, and inventory management functions?	
In progress No Yes	18 (22.2%) 22 (27.2%) 41 (50.6%)
Has the pharmacy estimated the quantities of essential patient care medications, materials and equipment, and PPE that would be needed during the pandemic phase?	
In progress No Yes	23 (28.4%) 7 (8.6%) 51 (63.0%)
Has the pharmacy made a plan to ensure the availability of essential patient care medications, materials and equipment, and PPE needed during the lockdown?	
In progress No Yes	25 (30.9%) 5 (6.2%) 51 (63.0%)
Has the pharmacy developed a list of alternative suppliers for essential medications and devices and a strategy to address shortages?	
In progress No Yes	31 (38.3%) 9 (11.1%) 41 (50.6%)
Has the pharmacy participated in the development of the hospital treatment plan/guidelines/order sets to treat COVID-19 positive patients?	
In progress No Yes	27 (33.3%) 16 (19.8%) 38 (46.9%)
Has the pharmacy documented critical pharmacy activities and identified essential pharmacy staff needed to perform them?	
In progress No Yes	26 (32.1%) 21 (25.9%) 34 (42.0%)
Has the pharmacy identified the minimum staffing needs and prioritized critical services based on essential hospital operations?	
In progress No Yes	18 (22.2%) 13 (16.0%) 50 (61.7%)
Has the pharmacy determined strategies for staffing, including tele-pharmacy, remote work, split shifts, etc.?	
In progress No Yes	22 (27.2%) 20 (24.7%) 39 (48.1%)
Has the pharmacy participated in hospital planning related to staff absences, employees’ inability to work—including illness, family issues, fear/anxiety?	
In progress No Yes	20 (24.7%) 24 (29.6%) 37 (45.7%)
Has the pharmacy or the hospital developed guidance for staff monitoring for signs of illness and created mechanisms for reporting both illness and absenteeism?	
In progress No Yes	23 (28.4%) 8 (9.9%) 50 (61.7%)
Has the pharmacy or hospital developed a return to work post-illness policy for employees?	
In progress No Yes	19 (23.5%) 30 (37.0%) 32 (39.5%)
Has the department of pharmacy identified points of contact for COVID-19 pandemic planning resources within the hospital?	
In progress No Yes	23 (28.4%) 25 (30.9%) 33 (40.7%)

## Discussion

Pharmacists are front line responders for COVID-19 patient care [27]. In this study, the authors assessed the knowledge, attitude, practices, and preparedness of a representative sample of hospital pharmacists towards COVID-19 pandemic across Lebanon. Knowledge of hospital pharmacists regarding COVID-19 is important. Hospital pharmacists are essential members of inter-professional health care teams especially during outbreaks, where they serve a vital role in infection control and in managing the medication therapies of patients hospitalized due to COVID-19 or related illnesses [28, 29]. Moreover, providing drug information is one of the main professional responsibilities of all pharmacists [30]. As they are assuming their advisory role on the use of pharmaceutical products, hospital pharmacists should also be aware of the recommendations concerning masks for example [31]. The majority of our study respondents showed appropriate knowledge regarding COVID-19 methods of transmission, preventive measures, and medications used. This finding is consistent with many studies that surveyed hospital pharmacists in different countries (Turkey, Vietnam, and Pakistan) and showed adequate knowledge about COVID-19 [32–34].

In mid-February, the WHO announced that COVID-19 outbreak was accompanied by an “infodemic” defined as an overabundance of information, making it highly prone to falsehoods and misinformation [35]. Consequently, healthcare providers should carefully evaluate COVID-19 information sources and utilize trustable resources to seek information [36, 37]. Most of the hospital pharmacists in our study reported using trusted resources from official websites including websites of MOPH, CDC, WHO, and Infectious Diseases Society of American (IDSA). Around half of the pharmacists also reported using television as a source of information. Different surveys in the USA and UK showed that television viewing during March and April 2020 has remarkably surpassed previous years, where public service media are viewed as a trusted source of information on the COVID-19 crisis [38–40]. Around one-third of pharmacists reported using Facebook for COVID-19 information, which is an unsafe practice. A new report has found that dozens of popular Facebook pages are publishing, repeating, and sharing false stories about the new coronavirus, related to conspiracy theories and false cures [41].

Concerning the attitude of Lebanese hospital pharmacists during the COVID-19 pandemic, most of the respondents reported exhaustion and concerns about being infected due to their professional exposure. Respondents, however, claimed that their stress did not affect their professional duties. Fear and exhaustion are common complaints among healthcare providers across the globe as the COVID-19 pandemic has taken a heavy toll on them [42].

The majority of the hospital pharmacists surveyed reported positive practices regarding safety and protective measures from COVID-19 infection, but reported shortages or delays in the supply of masks, gloves, and hand gels. The provision of personal protective equipment items and other medical devices should be adequately ensured in hospitals as they are used every day to provide adequate protection for healthcare professionals and patients as needed [31, 43]. The Chinese Pharmaceutical Association compiled a list of key facilities, equipment, and personal protective equipment of COVID-19 infections, particularly to hospital pharmacy settings [44]. As the number of affected countries and infected patients increased, the world faced a global shortage leading to disruption or delay of supplies [45]. In response to these threatening shortages, CDC and other non-official organizations recommended several strategies to optimize the supply and use of these equipment items and devices [43, 45, 46]. It is to note that the shortages in Lebanon were exacerbated by the harsh financial crisis and dollar shortage the country has been facing even before the COVID-19 pandemic [47, 48].

Along the same line, the WHO and the CDC have developed preparedness tools and checklists to enhance the readiness of healthcare professionals and facilities responding to COVID-19 [49, 50]. More specifically, the American Society of Health-System Pharmacists (ASHP) and the International Pharmaceutical Federation (FIP) has issued guides for health-system pharmacy departments and the pharmacy workforce respectively, to assist in planning efforts of the pharmacy department and to foster pharmacist involvement at the institutional and community levels in the fight of COVID-19 [17, 31]. These guides mainly focused on the role of the hospital pharmacist in ensuring adequate stock of medications and supplies, planning proper staffing needs, and providing adequate professional education and training. In this study, we surveyed participants between March 29 and May 10, 2020, 2 weeks to 2 months after the WHO declared COVID-19 outbreak a global pandemic [51]. Around two thirds of the surveyed pharmacists reported taking practical steps in their departments in preparation for COVID-19 pandemic or progressively working on their plans. Around 30% of the respondents, however, reported not taking any measures. The lowest preparedness (around 55%) was reported for the designation of a pharmacy staff member to coordinate education and training on COVID-19. The unpreparedness of some hospitals could be attributed to the previously mentioned financial crisis in the country, which made it harder for Lebanese hospitals to cope [47, 48]. Another potential reason is the fact that the local COVID-19 health strategic preparedness and response plan developed by the Lebanese MOPH did not include detailed measures or preparedness plans for pharmacy departments. It rather mentioned the importance of ensuring healthcare service continuity through maintaining adequate supplies of pharmaceuticals and medical devices [52].

At the time the investigators started this study, the number of infected people in the country was around 400 [23]. It was around a week after the country’s prime minister declared a state of a medical emergency and announced the closure of the airport, seaports, and land entrances. As of June 1, the number of COVID-19 patients in the country reached around 1233 cases (around 180 cases per 1 million of the population) [2]. This makes Lebanon among the countries that successfully flattened the curve, as the numbers were much lower than previously projected [53, 54]. Currently, Lebanon is preparing for a gradual exit from the lockdown [55]. This is happening amidst fears from a second wave, as the numbers of infected people per day are increasing again [23]. This requires continuous readiness for the healthcare system.

To our knowledge, this is the first study that has assessed the knowledge, attitude, practice, and preparedness of hospital pharmacists regarding COVID-19 in Lebanon. Potential limitations include the relatively small sample size. In addition, as the survey was in English language, Arabic/French speaking pharmacists may have refrained from filling it due to language barrier. This may have contributed to the relatively small sample size. Concerning the sample representability, the distribution of hospital pharmacists in Lebanon per geographical area is as follows: Bekaa (10.6%), South (13.4%), North (12%), Beirut (35.3%), and Mount Lebanon (28.6%). Therefore, we can conclude relatively lower representability of hospitals from the south area (a remote region from the capital), suggesting a probable overestimation of the results we obtained. Finally, there is a possibility of information bias, as surveys were self-administered and some pharmacists may have understood some questions differently. Further studies are suggested to take into account these weaknesses; nevertheless, we have no reason to believe that this would change the main results we obtained.

## Conclusion

Our findings revealed an appropriate level of knowledge and good practices towards COVID-19 among respondents from Lebanese hospital pharmacists. However, hospital pharmacists have not been engaged by the ministry of public health in the health emergency plans. This translated on delaying hospitals preparedness as a whole. National officials and organizations may benefit in utilizing the expertise of the hospital pharmacists to be able to minimize/avoid future waves of COVID-19 and mitigate the risk if these waves emerge.

## Supplementary information

**Additional file 1.** Questionnaire 486 COVID-19 Hospital Pharmacist Survey

## Data Availability

There is no public access to all data generated or analyzed during this study to preserve the privacy of the identities of the individuals. The dataset that supports the conclusions is available to the corresponding author upon request.
