# Supplementary material for: Assessing knowledge, attitude, practice, and preparedness of hospital pharmacists in Lebanon towards COVID-19 pandemic: a cross-sectional study
Source: J Pharm Policy Pract. 2020 Sep 18;13:54. doi: 10.1186/s40545-020-00266-8 (PMC7498300; doi:10.1186/s40545-020-00266-8)
Supplement: Supplementary file 1 — Additional file 1. Questionnaire 486 COVID-19 Hospital Pharmacist Survey [file 40545_2020_266_MOESM1_ESM.docx]

**Additional file 1: Questionnaire**

**COVID-19 Hospital Pharmacist Survey**

Date of filling the questionnaire: _____________

**SECTION I: GENERAL DEMOGRAPHICS ,**

1. **Age**: _______ years
2. **Sex:**

Male Female

1. **Years of experience in hospital pharmacy practice:**  _______years
2. **Please select your educational level degrees/certifications? (Check all that apply)**

BS Pharmacy Doctor of Pharmacy (Pharm.D./D.Pharm)

Masters (MS, MPH, MBA) PhD

Board Certifications (BCPS, BCOP, BCACP, BCSCP, etc… ) Others

1. **Job title / Position**

Director of Pharmacy/Chief Associate/Assistant Director/Chief

Clinical/Staff Pharmacist

1. **Status**

Part time Full Time

1. **Please select the location (Mohafaza) of the hospital you are working in:**

Beirut Mount Lebanon North South Bekaa

1. **Please select the one category that best describes the location of your hospital:**

Urban/City Rural/Peripheral Area

1. **Please select the one category that best describes the number of inpatient beds in your hospital:**

Up to 25 beds

26-99 beds

100-199 beds

200-299 beds

300-499 beds

500 beds and over

1. **Please select the one category that best describes the type of organization:**

Private – University Medical Center (UMC)

Private – Not UMC

Government – UMC

Government – Not UMC

1. **How many staff/clinical pharmacists are on duty at any single point in time at the pharmacy department?**
2. 1
3. 2
4. 3
5. 4 or more
6. **How many technicians are on duty at any single point in time at the pharmacy department?**
7. 1
8. 2
9. 3
10. 4 or more
11. **Does your facility have a physician residency-training program?**

No

Yes. Specify the affiliated university: ____________________

1. **Does your facility serve as a clinical site to train students from an accredited program?**

No

Yes. Select all that apply:

Medical students Registered nursing students Pharmacy students

Other: (please specify) _________________________

**SECTION II: KNOWLEDGE**

1. **Do you have time to get information regarding Covid-19 outbreak?**

3-4 hours/day 1-2 hours/day <1 hour/day Not at all

1. **Where do you get your information on COVID-19 from? (Check all that applies)**

- CDC website
- Ministry of Public Health website
- World Health Organization
- Infectious Disease Society of America
- Media website/Internet
- Facebook
- Friends/family members
- Television
- Other, specify: ……………………………

1. **Did you try to reach for the MOPH Coronavirus call center?**

No

Yes

1. **Were you able to contact the MOPH call center/hotline when you need it?**

No

Yes

1. **If you called the MOPH hotline/call center, how useful was the information provided:**

Very useful Somehow useful Not useful Not applicable

1. **Did you contact any other institution for supportive information on COVID-19?**

No

Yes

1. **If you other institutions for supportive information, how useful was the information provided:**

Very useful Somehow useful Not useful Not applicable

1. **Did you attend any awareness session on COVID-19?**

No

Yes

1. **Which of the following is true about COVID-19? (check all that apply)**

- Person to person transmission can occur by droplets
- Transmission can be airborne
- Most common signs and symptoms include fever, diarrhea and dyspnea
- I do not know

1. **What are the steps to take to protect yourself? (check all that apply)**

- Wash your hands with soap and water for at least 10 seconds
- Wash your hands with soap and water for at least 20 seconds
- Avoid close contact; put distance between yourself and other people (1.5-2 meters)
- Wear a facemask and stay home if you have any respiratory symptom
- No need to clean and disinfect solid objects (tables, doorknobs, desks, phones, etc.)

1. **Which of the below products are you recommending to patients to disinfect? (Check all that apply)**
2. Alcohol 60%
3. Alcohol 70%
4. Alcohol 95%
5. **Indicate which of the following options can be used to treat COVID 19 to date?**

- Acetaminophen
- Non-steroidal anti-inflammatory drugs (NSAIDS)
- Corticosteroids
- Symptomatic respiratory relief (inhalers)
- Lopinavir/ritonavir (initially for HIV)
- Chloroquine/remdesivir in combination
- Tocilizumab (initially for rheumatoid arthritis)

1. **Intravenous high dose vitamin C has been recommended in the treatment of COVID 19?**
2. True
3. False
4. I don’t know

**SECTION III: ATTITUDE**

**Kindly answer using a scale of Never to Always**

| **Questions** | **Never** | **Rarely** | **Often** | **Always** |
| --- | --- | --- | --- | --- |
| 1. Are you afraid of getting infected with COVID-19 due to occupational exposure? |  |  |  |  |
| 1. Are you afraid your family members get infected because of your occupational exposure? |  |  |  |  |
| 1. Do you feel depressed/exhausted due to the current pandemic? |  |  |  |  |
| 1. Are stress feelings affecting your duties (counseling, education, assessment)… |  |  |  |  |
| 1. Are stress feelings affecting your relationship with your staff and family members? |  |  |  |  |
| 1. Does any of your staff declare wanting to leave work due to COVID-19 fear? |  |  |  |  |
| 1. Do you implement specific icebreaking or energizing actions in your pharmacy to mitigate your staff stress? |  |  |  |  |

**Section IV: Practice - Safety of Pharmacy Staff**

| **Questions** | No | Yes | N/A |
| --- | --- | --- | --- |
| 1. Was any of your staff members infected by COVID-19? |  |  |  |
| 1. Are you required to wear a mask while performing your job at the pharmacy |  |  |  |
| 1. Are you required to wear gloves while performing your job at the pharmacy |  |  |  |
| 1. Are you wearing goggles/glasses to protect your eyes while performing your job at the pharmacy |  |  |  |
| 1. Are you still working as a full team as before COVID-19? |  |  |  |
| 1. If you are alternating schedule, were you asked to use vacation days? |  |  |  |
| 1. Did the working hours decrease for pharmacist/staff to decrease exposure? |  |  |  |
| 1. Did the working hours decrease for Director of Pharmacy/Chief Pharmacist to decrease exposure? |  |  |  |
| 1. In case your hospital serve as a training site for pharmacists, were training hours suspended during the pandemic? |  |  |  |
| 1. Did you make rotation for your staff to decrease exposure? |  |  |  |
| 1. Are you facing any shortage of masks, gloves or hand gels at your hospital? |  |  |  |
| 1. Are you facing any delay in the supply of masks, gloves or hand gels from the suppliers? |  |  |  |
| 1. Are you facing an increase in the price of the masks, gloves and hand gels from the supplier in a regular manner? |  |  |  |
| 1. Are you facing any pressure from the suppliers to pay on cash or in a short period of time? |  |  |  |

| 1. For how long do you put your face mask before changing it? (in hours) |  |
| --- | --- |
| 1. For how long do you put your gloves before changing them? (in hours |  |

**Kindly answer using a scale of Never to Always**

| **Questions** | **Never** | **Rarely** | **Often** | **Always** |
| --- | --- | --- | --- | --- |
| 1. Are you able to wash your hands during your work shift? |  |  |  |  |
| 1. Are you able to rub your hands with hydro-alcoholic gel during your work shift? |  |  |  |  |
| 1. Are you able to maintain social distancing of at least 1.5m from work colleagues |  |  |  |  |
| 1. Are you able to avoid touching eyes, nose and mouth? |  |  |  |  |
| 1. Are you able to stay home if you are not feeling well? |  |  |  |  |
| 1. Do you gloves during your work shift? |  |  |  |  |
| 1. Do you wear a mask during your work shift? |  |  |  |  |

**SECTION V: COVID19 PANDEMIC PLANNING EFFORTS AND PREPAREDNESS**

| **Questions** | **No** | **In Progress** | **Yes** |
| --- | --- | --- | --- |
| **Integration with Institutional Planning** | | | |
| 1. Is your hospital part of the national network of hospitals to receive Covid-19 positive patients |  |  |  |
| 1. Does your hospital have an emergency preparedness/management committee for COVID19? |  |  |  |
| 1. Is the pharmacy represented on the institution-wide emergency preparedness/management committee, and has it participated in the development of the institutional plan? |  |  |  |
| 1. Is the pharmacy familiar with best practices from other available plans (e.g., national - MOPH- or international – WHO, CDC) as they relate to pharmacy? |  |  |  |
| 1. Has the pharmacy participated in institutional development of an infection control plan for managing hospital patients with COVID-19? |  |  |  |
| **Departmental Leadership** | | | |
| 1. In the pharmacy plan, has the pharmacy included a section that describes backup for key pharmacy roles? |  |  |  |
| 1. Has the pharmacy designated a pharmacy department staff member to coordinate education and training on COVID-19? |  |  |  |
| **Public and Professional Education and Training** | | | |
| 1. Has the pharmacy prepared a plan to educate pharmacy staff on infection control measures, social distancing practices, personal protective equipment, prophylaxis, and treatment? |  |  |  |
| 1. Does the pharmacy routinely monitor to ensure that pharmacy staff adheres to infection control and social distancing measures? |  |  |  |
| 1. Has the pharmacy identified and trained multiple staff members to perform the critical pharmacy activities including administrative, clinical, distribution, and inventory management functions? |  |  |  |
| **Medications and Supplies** | | | |
| 1. Has the pharmacy estimated the quantities of essential patient care medications, materials and equipment, and personal protective equipment that would be needed during the pandemic phase? |  |  |  |
| 1. Has the pharmacy made a plan to ensure availability of essential patient care medications, materials and equipment, and personal protective equipment that would be needed during the waves? |  |  |  |
| 1. Has the pharmacy developed of a list of alternative vendors for essential medications and devices, and a strategy to address shortages? |  |  |  |
| 1. Has the pharmacy participated in the hospital treatment plan/guidelines/order sets to treat COVID-19 positive patients? |  |  |  |
| **Staffing** | | | |
| 1. Has the pharmacy documented critical pharmacy activities and identified essential pharmacy staff needed to perform them? |  |  |  |
| 1. Has the pharmacy identified the minimum staffing needs and prioritized critical services on the basis of essential institution-wide operations? |  |  |  |
| 1. Has the pharmacy determined strategies for staffing, including tele-pharmacy, remote work, split shifts, etc.? |  |  |  |
| 1. Has the pharmacy participated in hospital planning relating to staff absences and employees’ ability to work, including illness, family issues, fear/anxiety, etc.? |  |  |  |
| 1. Has the pharmacy (or the hospital) developed guidance for staff monitoring for signs of illness (including self-reporting, self-quarantine, and start/end of shift evaluation) and created mechanisms for reporting both illness and absenteeism? |  |  |  |
| 1. Has the pharmacy (or the hospital) developed a return to work post-illness policy for employees? |  |  |  |
| **Public Affairs / Communications** | | | |
| 1. Has the department of pharmacy identified points of contact for COVID-19 pandemic planning resources within the institution? |  |  |  |

Comment Section: if you have any comment, please share it with us.

______________________________________________________________________________________________________
